# Restoration of antibacterial activity of inactive antibiotics via combined treatment with a cyanographene/Ag nanohybrid

**DOI:** 10.1038/s41598-022-09294-7

**Published:** 2022-03-25

**Authors:** Lucie Hochvaldová, David Panáček, Lucie Válková, Robert Prucek, Věra Kohlová, Renata Večeřová, Milan Kolář, Libor Kvítek, Aleš Panáček

**Affiliations:** 1 Department of Physical Chemistry, Faculty of Science, Palacký University in Olomouc, 17. listopadu 12, 771 46 Olomouc, Czech Republic; 2 Regional Centre of Advanced Technologies and Materials, Czech Advanced Technology and Research Institute, Palacký University in Olomouc, Křížkovského 511/8, 779 00 Olomouc, Czech Republic; 3 Department of Microbiology, Faculty of Medicine and Dentistry, Palacký University in Olomouc, Hněvotínská 5, 775 15 Olomouc, Czech Republic

**Keywords:** Antibiotics, Antimicrobial resistance, Synthesis of graphene, Synthesis of graphene, Nanoparticles, Two-dimensional materials, Synthesis and processing

## Abstract

The number of antibiotic-resistant bacterial strains is increasing due to the excessive and inappropriate use of antibiotics, which are therefore becoming ineffective. Here, we report an effective way of enhancing and restoring the antibacterial activity of inactive antibiotics by applying them together with a cyanographene/Ag nanohybrid, a nanomaterial that is applied for the first time for restoring the antibacterial activity of antibiotics. The cyanographene/Ag nanohybrid was synthesized by chemical reduction of a precursor material in which silver cations are coordinated on a cyanographene sheet. The antibacterial efficiency of the combined treatment was evaluated by determining fractional inhibitory concentrations (FIC) for antibiotics with different modes of action (gentamicin, ceftazidime, ciprofloxacin, and colistin) against the strains *Escherichia coli*, *Pseudomonas aeruginosa,* and *Enterobacter kobei* with different resistance mechanisms. Synergistic and partial synergistic effects against multiresistant strains were demonstrated for all of these antibiotics except ciprofloxacin, which exhibited an additive effect. The lowest average FICs equal to 0.29 and 0.39 were obtained for colistin against *E. kobei* and for gentamicin against *E. coli*, respectively. More importantly, we have experimentally confirmed for the first time, that interaction between the antibiotic's mode of action and the mechanism of bacterial resistance strongly influenced the combined treatment’s efficacy.

## Introduction

Misuse and overuse of antibiotics has led to the rapid emergence of bacterial resistance to antibiotics following their introduction^1^. As a result, we are entering the so-called “post-antibiotic era” in which some bacterial infections risk becoming untreatable as they were in the past. In the US alone, 2.8 million people are infected by antibiotic-resistant bacteria every year, and about 35,000 of them die as a result^2^. The US centres for disease control and prevention (CDC) states that constantly increasing tendency of emerging bacterial resistance can be prevented by adopting a strategy concentrating on preventing infection and transmission, effective treatment, and diagnosis-based antimicrobial usage.

Current antibiotics cannot treat all known resistant bacterial infections, so there is a need for new antibiotics that should ideally target cellular pathways that microbes cannot readily modify. However, established drug development pathways have only yielded modifications of known antibiotic classes rather than the discovery of new ones. As a result, the number of newly developed antibiotics has decreased steadily over the last three decades and there is now a lack of effective treatment options for multidrug resistant infections. Additionally, inventing new drugs seems unattractive because of the high cost of research, the time-consuming drug approval process, the rather short window during which a new treatment remains effective and an effort to limit the using of antibiotics according to the principles of Antibiotic Stewardship^3–7^, including shortening the duration of antibiotic application. Because of these factors, many pharmaceutical companies are leaving the antimicrobial field or struggling to stay in business^8^.

Because new antibiotics seem unlikely to provide a way of quickly overcoming bacterial resistance in the near future, we need alternative ways of overcoming bacterial resistance. A very promising option is combination therapy, in which traditional antibiotics are combined with other substances that enhance their effectiveness. Bacteria can build up resistance towards certain antibiotics in various ways; consequently, antibiotics should ideally be applied in conjunction with a substance that can block the appropriate resistance mechanism. For example, because resistance to penicillins is driven by the production of β-lactamases, the efficacy of these antibiotics could be restored by applying them in tandem with β-lactamase inhibitors such as clavulanic acid, tazobactam, and sulbactam^9^. However, bacteria have since developed resistance even to these combined treatments, so there is a need for other options, preferably involving a complementary antibacterial agent capable of acting on multiple cellular levels simultaneously. Inorganic nanoparticles of materials such as silver, TiO_2_, ZnO, or graphene may be appropriate for this purpose; they exhibit strong antibacterial activity at very low concentrations (in the ppm range) and show no cytotoxicity towards various mammalian cell lines (human skin fibroblasts BJ or mouse embryonic fibroblast NIH/3T3 cell lines)^10–12^. Since antibiotics and nanoparticles have different modes of action, a combined treatment using low doses of both agent types could be highly effective. For example, it has been shown that treatment with silver nanoparticles (sometimes even at concentrations below 1 mg/L) can restore the susceptibility of resistant strains to antibiotics that are otherwise ineffective^11–15^. However, several recent publications have reported the development of bacterial resistance to even strong antibacterial agents such as silver nanoparticles^16–18^. For instance, Panacek et al. described a mechanism of bacterial resistance to silver nanoparticles based on aggregation induced by secretion of the bacterial protein flagellin. To maintain the strong antibacterial effectiveness of nanoparticles, their aggregation must be prevented. This can be achieved to some extent by surface modification and stabilization, but such approaches are unfortunately insufficient in most cases. Aggregation can be also suppressed by using nanocomposite materials that provide a supporting structure for strongly bonded silver nanoparticles and thereby suppress their aggregation^16^.

Graphene and its derivatives are an interesting class of supporting materials with promising antibacterial properties. For instance, graphene oxide (GO) exhibits antibacterial activity at concentrations as low as 40 mg/L, potentially making it suitable for medicinal applications. However, its strong inter-plane interactions promote aggregation, reduce the surface area of nanoparticles, and block certain antibacterial modes of action, leading to reduced antibacterial activity^19–21^. The aggregation of GO and the associated reduction in antimicrobial activity could be minimised by surface modification or functionalisation with metals, antibiotics, enzymes, or polymers^22^. For example, GO can be modified with silver nanoparticles^23,24^, whose deposition on a graphene surface may suppress their aggregation and improve their antibacterial properties. Accordingly, Panacek et al.^25^ recently reported the synthesis of cyanographene/Ag nanoparticles and showed that they exhibit highly promising activity against bacteria.

Given the strong antibacterial activity of cyanographene decorated with silver nanoparticles and the fact that antibiotics and silver nanoparticles are known to exhibit synergistic antibacterial activity, it was hypothesised that the minimal inhibitory concentrations of antibiotics when applied together with the cyanographene/Ag composite would be below the susceptibility breakpoints of resistant bacteria, making their effectiveness against resistant strains comparable to that seen for sensitive strains^11^. Therefore, this work investigates the synergistic antibacterial effects resulting from combining a cyanographene/Ag nanohybrid with inactive antibiotics having different modes of action (gentamicin, ceftazidime, ciprofloxacin and colistin) against bacteria with different resistance mechanisms. The objective was to find the combinations of antibiotics and the cyanographene/Ag nanohybrid exhibiting the greatest activity against the resistant strains and to thereby identify effective ways of overcoming bacterial resistance for individual antibiotics against particular resistant bacteria based on their mode of action and mechanism of resistance, respectively.

## Methods

### Chemicals and biological materials

The cyanographene/silver (GCN/Ag) nanohybrid was synthesized using fluorinated graphite (extent of labelling: > 61 wt% F), ammonia (28–30% [w/w], p.a.), sodium borohydride, and sodium citrate dihydrate (p.a.), all obtained from Sigma-Aldrich. Mueller–Hinton Broth (Becton, Dickson, and Company) was used as the culture medium. Synergetic effects were tested for combinations of the GCN/Ag nanohybrid with the antibiotics gentamicin (GEN), ceftazidime (CTZ), ciprofloxacin (CIP) against the multiresistant strain *Escherichia coli* CE5556. Resistance mechanisms of *Escherichia coli* CE5556 to β-lactams, quinolones and aminoglycosides described within this work were previously studied and confirmed by Roderova et al.,^26^ who identified cefotaximase-Munich CTX-M-15 type of extended spectrum β-lactamases and mutation of the gyrA gene [Ser (83) Leu; Asp (87) Asn], parc [Ser (80) Ile; Glu (84) Val], PMQR [cr] resulting in changes in the target enzyme DNA gyrase causing resistance to fluoroquinolones. Additional antimicrobial tests were performed against *Pseudomonas aeruginosa* 21,425 using combinations of GEN, CIP and CTZ with GCN/Ag; this strain shows similar resistance to antibiotics like *E. coli* CE 5556 as it was confirmed by phenotypic methods according to EUCAST^6^. Finally, tests were performed using colistin (COL) with GCN/Ag against *Enterobacter kobei* 3683/C/2017. Resistance to colistin was confirmed by phenotypic methods according to EUCAST^27^. The zeta potential of the sensitive and resistant strains (according to their MIC) was measured and a significant drop (from − 30 to − 5 mV) was observed, which has an effect on the interaction of the bacteria with positively charged colistin. All bacteria were obtained from the microorganism collection of the Department of Microbiology at the Faculty of Medicine of Palacky University in Olomouc. MIC (mg/L) values of used strains are shown in supplementary material (SI Fig. 4).

### Synthesis and characterization of GCN/Ag nanohybrid

The GCN/Ag nanohybrid was prepared by synthesizing cyanographene and then coordinating silver NPs on the GCN sheet as described previously by Panacek et al.^25^. Briefly, GCN/Ag was synthesized by chemical reduction of a precursor material, then silver cations from AgNO_3_ were coordinated on the GCN sheet under vigorous stirring for 24 h at room temperature. The resulting dispersion of Ag^+^-modified GCN was purified by washing with distilled water to remove silver ions not firmly coordinated on the GCN flakes. Chemical reduction was then initiated by adding NaBH_4_ solution to the dispersion, which was then kept in darkness for one hour. The final silver-nanoparticle-decorated GCN/Ag product was washed with distilled water and dried. The GCN/Ag nanohybrid was characterised by various instrumental techniques; for details, see the supporting material.

### Determination of synergetic effects of antibiotics and AgNPs

A standard checkerboard microdilution method was used to determine the minimum inhibitory concentration (MIC) of the GCN/Ag nanohybrid and each antibiotic (ATB) by themselves and the MICs of different concentrations of nanohybrids in combination with different concentrations of antibiotics. The nanohybrid and antibiotics were always diluted in a geometric progression in Mueller–Hinton broth when determining MICs. Based on its strong antibacterial effect, the initial concentration of the GCN/Ag nanohybrid for microbial evaluation was set to 13.5 mg/L. The initial concentrations of the antibiotics depended on their reported susceptibility breakpoints and are shown in Table 1. Both nanoparticles and antibiotics were diluted in geometric progressions; the tested ATB concentrations were 256, 128, 64, 32, 16, 8, 4, and 2 mg/L for those with high initial concentrations and 1, 0.5, and 0.25 mg/L for those with lower initial concentrations. Due to the strong antibacterial activity of the GCN/Ag nanohybrid, its initial concentration was lower; the dilution series began at 3.375 mg/L (based on the mass of silver) and progressed to 1.688, 0.844, 0.422, 0.211, 0.105, and 0.002 mg/L. To determine the enhancement of antibacterial activity resulting from combined treatment with GCN/Ag and the studied antibiotics, 96-well microtitration plates were filled with vertically diluted antibiotics and horizontally diluted GCN/Ag.

**Table 1:** Stock solution concentrations of the tested antibiotics in mg/L.

	*E. coli*	*P. aeruginosa*	*E. kobei*
CIP	512	256	–
CTZ	1024	256	–
GEN	1024	128	–
COL	–	–	256

For each antimicrobial assay, a fresh bacterial suspension was prepared from bacteria that had been grown on blood agar at 35 °C for 24 h. The optical density of the bacterial inoculum was determined to be equal to 1 based on McFarland´s standard using a densitometer (Densi-La-Meter, LACHEMA, Czech Republic); after appropriate dilution, this gave a starting concentration of 10^6^ CFU for microbial testing. Antibacterial activity was assessed according to standard testing protocols (CLSI, EUCAST) and the MIC was determined as the lowest concentration of antibacterial agent that visibly inhibited bacterial growth after 24 h incubation at 35 °C.

The fractional inhibitory concentration (FIC) index was calculated using the following equation:

$$ \overline{{{\text{FIC}}}} = \frac{{MIC_{Ag } \;in\; combination}}{{MIC_{Ag} \; alone}} + \frac{{MIC_{ATB } \;in\; combination}}{{MIC_{ATB} \; alone}} $$

Results are reported as average $\overline{{{\text{FIC}}}}$ values (calculations shown in SI); antibacterial effects are classified as synergistic (FIC ≤ 0.5), partially synergetic (0.5 ˂FIC ≤ 1), additive (FIC = 1), indifferent (1 ˂ FIC ˂ 4), or antagonistic (FIC ≥ 4)^15,28,29^.

## Results

**Figure 1:**
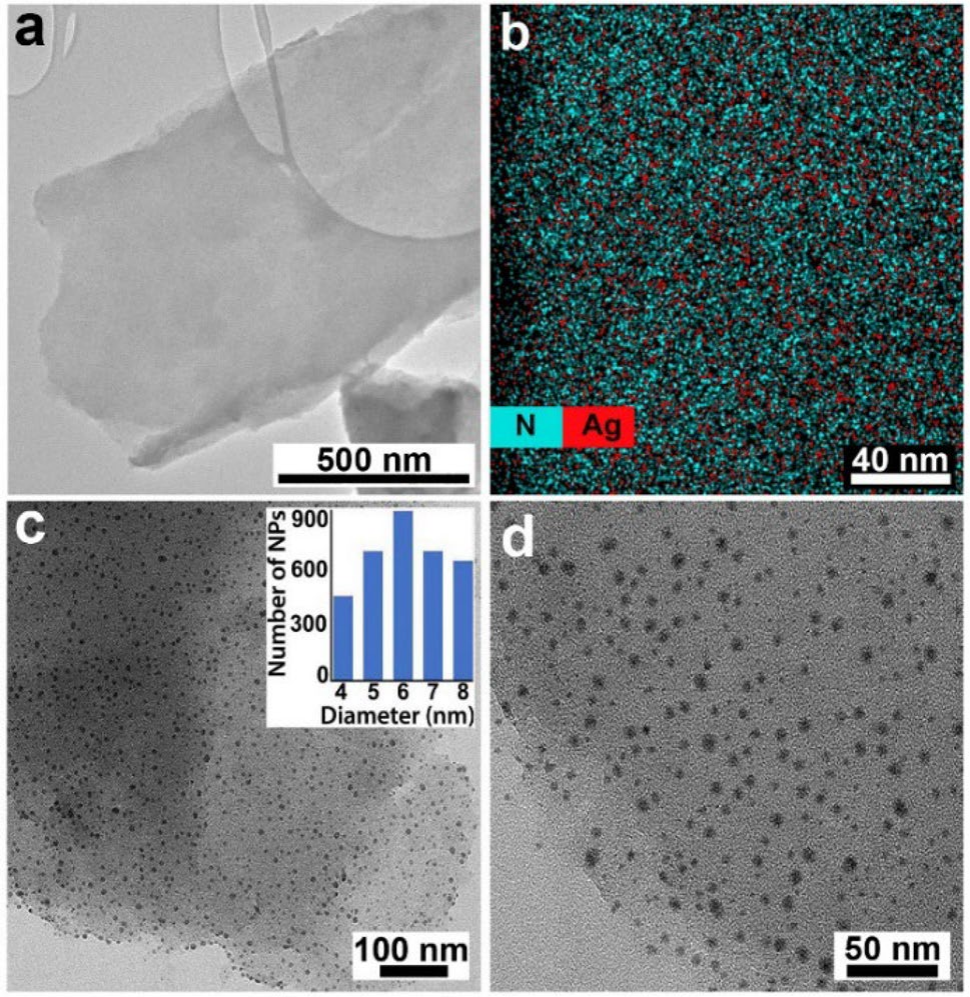
(**a**) HRTEM image of the GCN/Ag^+^ precursor. (**b**) Combined chemical mapping of nitrogen and silver on the graphene surface. (**c**,**d**) TEM images of GCN/Ag and size distribution of the AgNPs (inset in panel **c**).

A cyanographene/silver nanohybrid (GCN/Ag) was synthesized via a previously published method based on the reduction of Ag^+^ ions bound to a cyanographene derivative modified with nitrile groups referred to as the GCN/Ag^+^ precursor^25^. High-resolution transmission electron microscopy images of the GCN/Ag^+^ precursor (Fig. 1a) confirmed the absence of AgNPs prior to the application of the reducing agent. Furthermore, chemical elemental mapping revealed dense and homogeneous coverage of the graphene flakes with both nitrogen and silver atoms (Fig. 1b). After removing unbound silver ions by thorough washing, reduction with NaBH_4_ provided a final GCN/Ag product consisting of small AgNPs (Fig. 1c,d) with diameters of 4 to 8 nm (Fig. 1c, inset).

**Figure 2:**
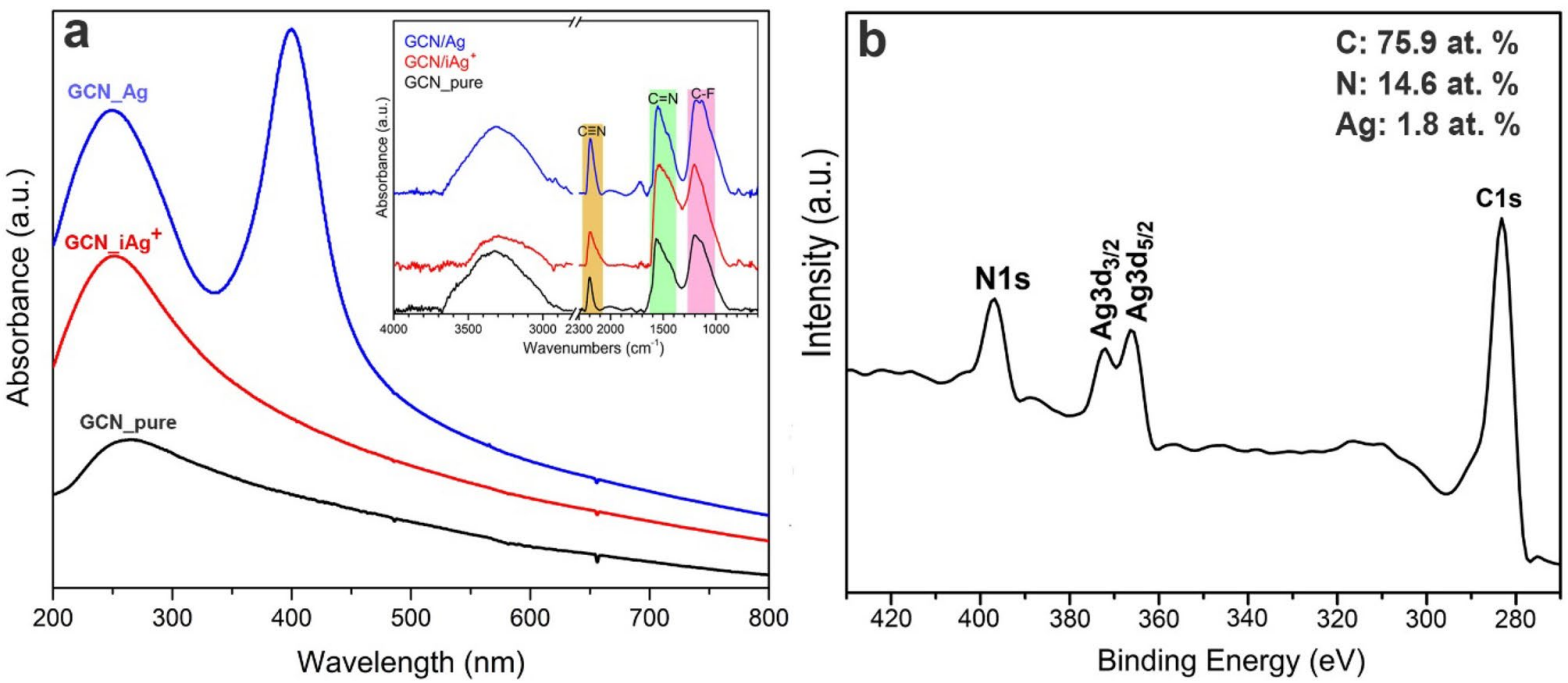
(**a**) UV–Vis absorption spectra of pure GCN (black line), GCN with immobilized ionic silver (red line), and GCN with immobilized AgNPs (blue line); the corresponding FT-IR spectra are shown in the inset of panel (**a**). (**b**) XPS survey spectrum of the GCN/Ag nanohybrid showing atomic content of the material.

Absorption spectrophotometry of the precursor GCN/Ag^+^ and GCN/Ag water dispersions confirmed that the nanoparticles formed only after treatment with the reducing agent (Fig. 2a) based on the appearance of the characteristic surface plasmon resonance of metallic AgNPs at a wavelength of 400 nm^30^. Nitrile groups were also observed by FT-IR before and after immobilization of AgNPs, confirming their stability even after AgNPs binding; the inset of Fig. 2a shows that the nitrile peak at around 1500 cm^−1^ is present in the spectra of both the precursor and the reduced product. The Ag content of the GCN/Ag hybrid was 1.8 at. % (atomic content), according to X-ray photoelectron spectroscopy (Fig. 2b). The exact amount of silver in water dispersions of GCN/Ag after purification before the antibacterial assays was 330 mg/L, corresponding to 125 mg of Ag per 1 g of GCN/Ag nanohybrid based on the AAS data.

The antibacterial activity of the purified GCN/Ag nanohybrid dispersion towards selected resistant bacteria was then tested both alone and in combination with various antibiotics. Note that all of the MICs reported for GCN/Ag in this work are based on the silver content of the GCN/Ag water dispersion (the silver concentration in the dispersion was 330 mg/L as determined by AAS). Table 2 summarises the minimum inhibitory concentrations (MICs) of the antibiotics (ATBs) ciprofloxacin (CIP), ceftazidime (CTZ), gentamicin (GEN), and colistin (COL) as well as the Ag-based MICs of the GCN/Ag nanohybrid against *E. coli, P. aeruginosa*, and *E. kobei*. The table also shows the MIC ranges of GCN/Ag and each ATB when applied in combination together with the lowest, highest, and mean fractional inhibitory concentrations (FICs) determined for each antibiotic/nanohybrid pair. Enhanced (i.e., synergistic or partially synergetic) antibacterial effects against resistant *E. coli* were observed for the combinations of GCN/Ag (Ag-related MIC: 1688 mg/L) with GEN, CTZ, and CIP. The strongest synergistic effect and enhancement of activity against *E. coli* was observed for gentamicin with GCN/Ag: this pairing yielded the lowest partial FIC (0.16) and average FIC (0.39). Combined treatment with GCN/Ag reduced the MIC of gentamicin 32-fold, to just 4 mg/L, and reduced that of the nanohybrid eightfold, to 0.211 mg/L. Four additional antibacterial combinations with low partial FIC index values (ranging from 0.19 to 0.31) indicating strong enhancement of activity against *E. coli* were also discovered (see Figure S1, Supplementary information). The highest FIC index value for the gentamicin/nanohybrid pair (0.53) was obtained when combining 64 mg/L gentamicin with 0.053 mg/L GCN/Ag (see Figure S1). While this FIC value corresponds to a partial rather than full synergistic effect, the cumulative FIC for gentamicin with the nanohybrid (defined as the arithmetic mean of all calculated FICs; see the SI for details) was 0.39, indicating a synergistic antibacterial effect. Partial synergy (FIC 0.54) was also observed for the combination of ceftazidime with GCN/Ag when used against *E. coli*. The enhancement in this case was weaker than for gentamicin (FIC 0.39), but even so, combining ceftazidime with the nanohybrid made it possible to achieve an MIC below the susceptibility breakpoint; the MIC of ceftazidime was reduced from 32 to 1 mg/L when applied together with 0.844 mg/L GCN/Ag. The lowest FIC observed for the ceftazidime/nanohybrid pair was 0.38, which was achieved when combining 8 mg/L ceftazidime with 0.211 mg/L GCN/Ag. The highest calculated FIC for this pair (0.63) was still within the partially synergetic range (Figure S1). The final antibiotic tested against *E. coli* was ciprofloxacin, which exhibited an additive effect (FIC = 1.00) when combined with the GCN/Ag nanohybrid, suggesting an indifferent effect without significant enhancement of activity. The only combination showing any potentially synergistic effect for this antibiotic featured intermediate concentrations of both agents − 32 mg/L ciprofloxacin and 0.844 mg/L GCN/Ag (Figure S1).

**Table 2:** Minimum inhibitory concentrations MIC [mg/L]^a^ of various antibiotics and the GCN/Ag nanohybrid, average $\overline{{{\text{FIC}}}}$^b^ values for combinations of antibiotics with the GCN/Ag nanohybrid, and the resulting antibacterial effects.

	*E. coli*			*P. aeruginosa*			*E. kobei*
	GEN	CTZ	CIP	GEN	CTZ	CIP	COL
ATB alone	128	32	64	8	8	16	64
GCN/Ag alone	1.688	1.688	1.688	1.688	1.688	1.688	3.375
ATB in combination	4–64	1–16	32	0.5–4	1–4	8	1–32
GCN/Ag in combination	0.003–0.844	0.211–0.844	0.844	0.105–0.844	0.844	0.844	0.422–1.688
Partial FIC	0.16–0.53	0.38–0.63	1.00	0.38–0.56	0.75–1.00	1.00	0.16–0.63
$\overline{{{\text{FIC}}}}$	0.39	0.54	1.00	0.53	0.88	1.00	0.29
Effect	(S)	(PS)	(A)	(PS)	(PS)	(A)	(S)

^a^The MIC determination is a qualitative assay and the estimated error is of the order of magnitude of the value; (n = 3).

^b^Average value (SD); (n = 3).

*(S) synergy, (PS) partial synergy, (A) additive.

**GCN/Ag shows silver related concentration.

***The ranges given in the *ATB in combination* row of this table indicate the ranges of MICs obtained for the indicated antibiotic when applied with varying concentrations of the GCN/Ag nanohybrid. Similarly, the ranges given in the *GCN/Ag in combination* row indicate the ranges of MICs obtained for the nanohybrid when applied in combination with varying concentrations of the indicated antibiotic.

In the case of resistant *P. aeruginosa*, the FICs of all antibiotics were slightly higher than for resistant *E. coli*, but the Ag-related MIC of GCN/Ag was the same (1.688 mg/L) as for *E. coli*. The average FIC for gentamicin (0.53) was in the range associated with partial synergy, but its lowest determined FIC (0.38) was in the synergistic range. In this case, combined treatment with just 0.422 mg/L GCN/Ag reduced the antibiotic concentration needed to get below the gentamicin breakpoint by a factor of eight. The partial FIC values determined for gentamicin combined with GCN/Ag were mostly in the synergistic effect range, with just two in the partially synergetic range, but this was sufficient to raise the average FIC for this combination into the partially synergetic range (Figure S2). Partial synergy against resistant *P. aeruginosa* was also observed for the pairing of ceftazidime combined with GCN/Ag, whose average FIC of 0.88 was higher than that of gentamicin (0.53) and also higher than that for ceftazidime when used against *E. coli* (0.54). However, even in this case, with partial FICs ranging from 0.75 to 1.00, combined treatment with 0.844 mg/L GCN/Ag reduced the ceftazidime concentration needed to kill resistant *P. aeruginosa* by a factor of four. The FIC index for the combination of ciprofloxacin with the GCN/Ag nanohybrid when tested against *P. aeruginosa* was 1.0, indicating an additive effect with no significant improvement in antibacterial activity; this is consistent with the results obtained for ciprofloxacin when used against resistant *E. coli.*

The MIC of the GCN/Ag nanohybrid against colistin-resistant *E. kobei* was 3.375 mg/L. Combined treatment with GCN/Ag and colistin yielded a strong synergistic effect with a very low FIC index of 0.29, overcoming the strain’s antibiotic resistance. Under optimal conditions, the combined treatment reduced the MIC of colistin 32-fold, down to just 2 mg/L when applied together with 0.422 mg/L GCN/Ag. This combined treatment also reduced the MIC of the nanohybrid eightfold and yielded a very low FIC of 0.16. Other combinations of colistin with GCN/Ag at concentrations of 1–16 mg/L and 0.422–0.844 mg/L, respectively, had partial FICs between 0.19 and 0.38, confirming a strong synergistic effect for this pairing (Figure S3). The highest FIC observed for the pairing of colistin with GCN/Ag against *E. kobei* was 0.63, which was seen when combining 32 mg/L colistin with 0.422 mg/L GCN/Ag.

## Discussion

A GCN/Ag nanohybrid was prepared according to a previously published protocol^25^ that involves cyanographene synthesis followed by coordination of silver NPs on the GCN sheet. Many previous studies have used graphene oxide (GO) as a solid support for the immobilization of AgNPs due to its ability to prevent their aggregation^31–33^. However, its surface is chemically inhomogeneous with many different oxygen-containing groups, which prevents strong and selective silver binding at the surface^34,35^. Furthermore, the hard soft acid base theory suggests that oxygen-containing functional groups are unsuitable ligands for silver binding^36^. To overcome these limitations, densely functionalized graphene (cyanographene, GCN)^37^ was used as a solid support and was shown to be a very effective covalent trap for silver ions due to the strong coordination of silver ions by nitrile groups^36^. This strong binding enabled the preparation of a highly pure GCN/Ag^+^ precursor and subsequent reduction of only those Ag ions that remained firmly anchored to the GCN support (Fig. 1a).

According to Panacek et al. and the results presented herein, dispersions of the cyanographene silver nanohybrid in water have ultralow MICs ranging from 1.8 to 14.7 mg/L. If MIC values are based solely on the silver content of the hybrid rather than its total mass, the MICs are lower still (up to 3.375 mg/L) and fall below those reported for 28 nm AgNPs dispersed in water (3.4–108 mg/l)^11,25^. The strong covalent immobilization and dense coverage of the graphene surface with AgNPs yielded a material with excellent properties that was recently used as a very effective antibacterial material against AgNPs-resistant bacterial strains^25^. Because the GCN/Ag nanohybrid has greater antibacterial activity than typically used silver nanoparticles, it was expected that it would also show strong antibacterial properties when combined with antibiotics. The aim of this work was to restore the antibacterial activity of antibiotics that have become ineffective against resistant strains. Reducing the MICs of antibiotics via combined treatment with GCN/Ag has important therapeutic advantages because it reduces the amount of the antibiotic and nanocomposite that must be applied to control bacterial infections, reducing the risk of adverse effects in patients. Moreover, the GCN/Ag nanohybrid shows little cytotoxicity in human embryonic lung fibroblasts (HEL), human skin fibroblasts (BJ) and human cervix adenocarcinoma cells (HeLa); it was fully tolerated at concentrations of 60 mg/L (based on the total mass of the composite) or 7.5 mg/L (based only on its silver content)^25^.

**Figure 3:**
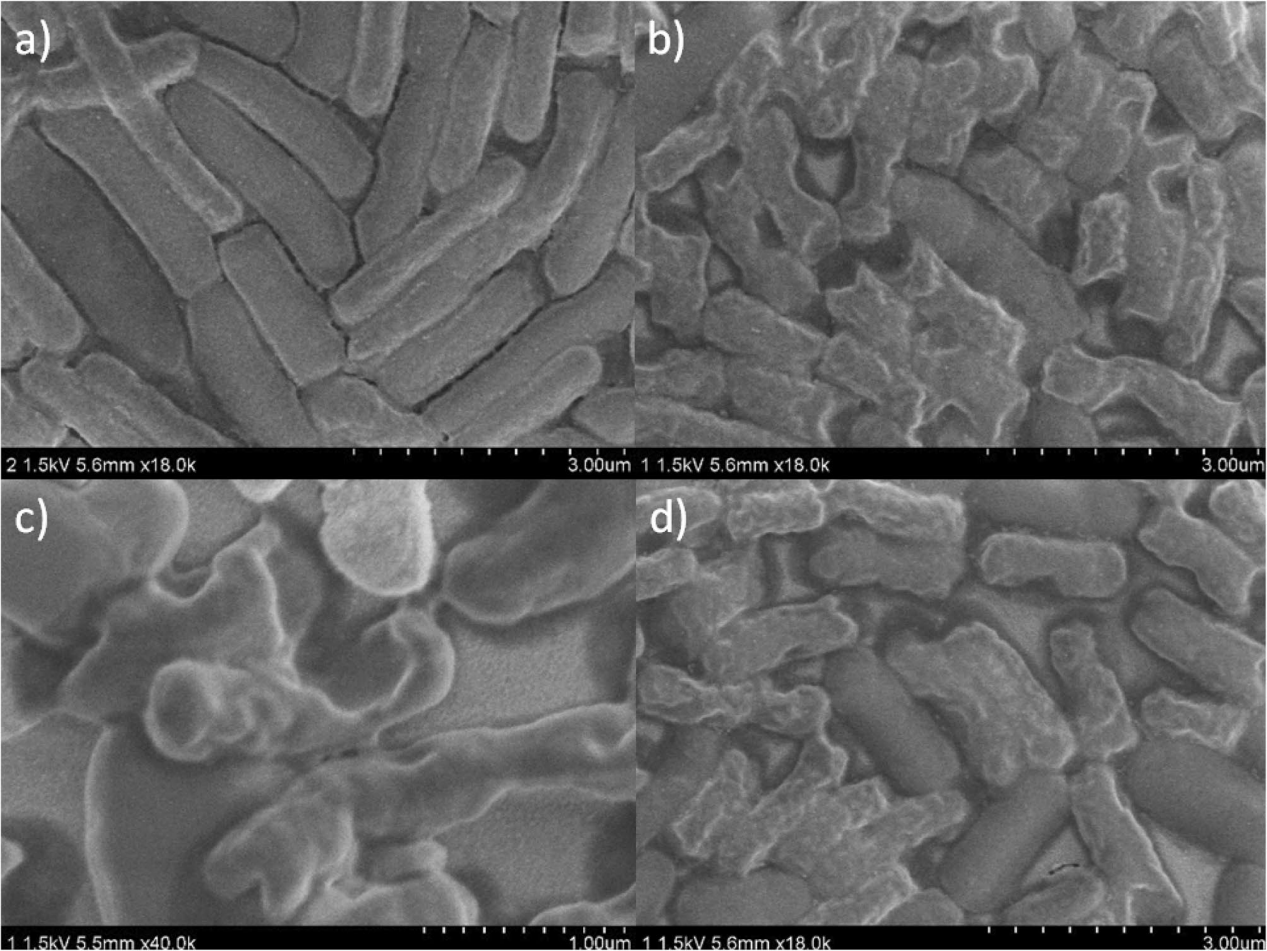
SEM images of (**a**) untreated *E. coli* and *E. coli* with disrupted cell walls after treatment with (**b**,**c**) GCN/Ag (bacteria on image (**c**) were captured at higher magnification), (**d**) GCN/Ag and gentamicin.

Each of the antibiotics tested in this work belongs to a different antibiotic class and interacts with bacteria via a different mechanism^38^. Gentamicin belongs to the group of aminoglycoside antibiotics, which inhibit protein synthesis by binding to the 30S subunit of the prokaryotic ribosome. The bacteria *E. coli* studied in this work resist gentamicin by reducing their cell permeability, which was overcome via the outer membrane- and cell wall-disrupting ability of the GCN/Ag nanohybrid (see Fig. 3). Disruption of the bacterial cell membrane and cell wall increases the permeability of the bacterial cells, allowing gentamicin to reach its intracellular target site.

Ceftazidime is an antibiotic whose mode of action is based on inhibiting cell wall synthesis. *E. coli* strains have developed resistance to its activity by producing extended spectrum β-lactamases^26^ that hydrolyse the beta-lactam core of these antibiotics, making them unable to bind to their usual target site. It is most likely that the nanohybrid counteracts this resistance by penetrating the outer membrane, allowing a more intense and easier release of β-lactamases in higher extent than in the case of a bacteria with an intact membrane and thereby weakening their negative effect on the antibiotics.

In contrast, colistin is an antibiotic that targets the inner cell membrane of bacteria by binding to key components of the cellular envelope (phospholipids and lipopolysaccharides) and displacing magnesium and calcium ions that stabilize the membrane. This increases the membrane’s permeability, leading to loss of cellular components and cell death^39^. The resistant *E. kobei* strain examined in this work exhibits colistin resistance in which the outer membrane is modified (has slight negative charge equal to—8 mV compared to—38 mV of sensitive strain) in a way that prevents the antibiotic (positively charged with zeta-potential value 20 mV) from reaching its target, i.e. the inner membrane. The enhancement of colistin’s antibacterial activity upon combined treatment with GCN/Ag is probably due to adsorption of the positively charged colistin (zeta potential: 20 mV) on the negatively charged GCN/Ag nanohybrid (zeta potential: − 35 mV) via electrostatic interactions. This would allow the nanohybrid to act as a nanocarrier of colistin, which may weaken the repulsive interactions between colistin and the outer membrane. Additionally, the nanohybrid may disrupt the bacterial outer membrane and cell wall, giving the antibiotic easier access to the inner membrane. In this case, although the mechanisms of action of the antibiotic and the nanohybrid are similar, their combination results in strong enhancement of antimicrobial activity.

The final antibiotic tested in this work was ciprofloxacin, which belongs to the quinolone class of antibiotics whose mechanism of action involves inhibiting nucleic acid synthesis by inhibiting DNA gyrase and the type II and type IV topoisomerases, which are crucial for bacterial DNA separation and cell division. Ciprofloxacin resistance in the tested *E. coli* strain originates from a DNA gyrase mutation^26^ that prevents proper binding of the antibiotic. Unlike the previously discussed resistance mechanisms, this one does not involve changes in the cell wall or membrane; instead, it affects the structure of a nuclear protein. Consequently, the nanohybrid has no chemical or biological way to affect this mechanism, so only additive rather than synergistic or partially synergetic effects were seen in this case.

The activity of a GCN/Ag nanohybrid in combination with various antibiotics has been tested against various antibiotic-resistant bacterial strains. The results obtained suggest that combined treatment enhances antibacterial activity in some cases but that the degree and nature of the enhancement depends on the underlying mechanism of resistance and the mode of action of the antibiotic. Promising results were obtained for an antibiotic that blocks bacterial protein synthesis by binding to the 30 s subunit of the bacterial ribosome (gentamicin) and for one that weakens cytoplasmic membrane integrity (colistin). Synergistic antimicrobial effects were observed when these antibiotics were applied in combination with the nanohybrid, causing their MICs to fall below their susceptibility breakpoints even against multiresistant bacterial strains. Importantly, even very low concentrations of the nanocomposite (usually 0.422 mg/L) were sufficient to restore bactericidal activity against *E. coli* and *P. aeruginosa*. Positive results were also obtained for ceftazidime, which acts by inhibiting bacterial cell wall synthesis. However, combined treatment with the nanohybrid did not greatly enhance the activity of ciprofloxacin, which acts by inhibiting bacterial DNA synthesis. The present findings help to better understand the mechanisms of combination antibacterial therapy using antibiotics together with other antimicrobials and open the way how to restore antibacterial efficiency of conventional antibiotics and overcome bacterial resistance.

## Supplementary Information

Supplementary Information.

## Data Availability

All data generated or analyzed during this study are included in this published article (and its Supplementary Information files).
