# Supplementary material for: Restoration of antibacterial activity of inactive antibiotics via combined treatment with a cyanographene/Ag nanohybrid
Source: Sci Rep. 2022 Mar 25;12:5222. doi: 10.1038/s41598-022-09294-7 (PMC8956642; doi:10.1038/s41598-022-09294-7)
Supplement: Supplementary file 1 — Supplementary Information. [file 41598_2022_9294_MOESM1_ESM.docx]

**Supplementary material for**

**Restoration of antibacterial activity of inactive antibiotics via combined treatment with a cyanographene/Ag nanohybrid**

Lucie Hochvaldová^1^, David Panáček^1,2^, Lucie Válková^1^, Robert Prucek^1^, Věra Kohlová^1^, Renata Večeřová^3^, Milan Kolář^3^, Libor Kvítek^1^ and Aleš Panáček^1,*^

^1^Department of Physical Chemistry, Faculty of Science, Palacký University in Olomouc, 17. listopadu 12, 771 46 Olomouc, Czech Republic

^2^Regional Centre of Advanced Technologies and Materials, Czech Advanced Technology and Research In-stitute, Palacký University in Olomouc, Křížkovského 511/8, Olomouc 779 00, Czech Republic

^3^Department of Microbiology, Faculty of Medicine and Dentistry, Palacký University in Olomouc, Hněvotínská 5, 775 15 Olomouc, Czech Republic

* Corresponding author: Aleš Panáček

address: 17. listopadu 12, Olomouc, 77146, Czech Republic

e-mail: ales.panacek@upol.cz

tel: +420585634427

fax: +420585634425

**Instrumentation.** HR-TEM images were obtained using a HR-TEM TITAN 60-300 microscope with an X-FEG type emission gun, operating at 300 kV. Scanning transmission electron microscopy high-angle annular dark-field imaging (STEM-HAADF) was used for elemental mapping on the products and for EDS analysis (energy-dispersive X-ray spectroscopy) while using FEI Titan HR-TEM microscope operating at 80 kV. GCN/Ag was characterized by transmission electron microscopy (TEM) using a JEM 2010 TEM instrument (Jeol, Japan). For all of those analysis, a droplet of nanocomposite (~0.1 mg/L) was deposited on a carbon-coated copper grid and dried at room temperature for 24 hours and then studied. Bacteria were after incubation observed alone or in the presence of GCN/Ag by scanning electron microscope (Hitachi SU6600) with acceleration voltage 1.5 kV. All bacteria were incubated for 24 h in 96-well microtiter plates (standard dilution method). Sub-inhibitory concentration was washed (3 times) from broth residues by centrifugation and redispersion in PBS and in water finally. Later on, they were dropped on gold-coated microscope glass slides, airdried and fixated by flame. Absorption and FTIR spectra were recorded on a Specord S 600 (Analytic Jena, Germany) and iS5 FTIR (Thermo Nicolet) spectrophotometer using the Smart Orbit ZnSe ATR accessory, respectively. In case of ATR, droplet of an ethanol was placed on the ZnSe crystal and dried. The spectra were acquired in nitrogen atmosphere at ATR accessory via summing 52 scans and applying baseline and ATR corrections. The amount of the immobilized silver was measured via atomic absorption spectroscopy (AAS) using ContrAA 600 with graphite furnace (Analytik Jena AG, Germany) equipped with a high-resolution Echelle double monochromator (spectral band width, 2 pm at 200 nm) and a xenon lamp as a continuum radiation source. For AAS characterization, GCN/Ag (0.01 mg/L was dissolved in 2 % w/w solution of nitric acid and sonicated for 10 minutes. High-resolution X-ray photoelectron spectroscopy (HR-XPS) was carried out with a PHI VersaProbe II (Physical Electronics) spectrometer using an Al K_α_ source (15 kV, 50 W). The obtained data were evaluated and deconvoluted with the MultiPak (Ulvac - PHI, Inc.) software package, while the spectral analysis process involved Shirley background subtraction and peak deconvolution using mixed Gaussian–Lorentzian functions.

**Supplementary Tables and Figures.**

**Figure S1**. Schematic illustrating *E. coli* growth (grey) on microplates in microdilution checkerboard assay. Minimum inhibitory concentration for each antimicrobial is determined (blue). FICs are calculated for various combinations antibiotic-GCN/Ag nanohybrid inhibiting bacterial growth and at the same time minimal FIC (green), maximal FIC (red) and average FIC (FIC_AVR_) are highlighted and the resulting effect is described (SYN – synergy, PSY – partially synergy, ADD – additive effect).

Example of FIC calculation for GEN-GCN/Ag in E. coli is shown below.

$$FIC=\frac{{MIC}_{GCN/Ag}in combination}{{MIC}_{GCN/Ag}alone}+\frac{{MIC}_{ATB}in combination}{{MIC}_{ATB}alone}$$

$$FIC=\frac{0.211}{1,688}+\frac{4}{128}=0.125+0.03125=0.15625≐0.1$$

$$\bar{FIC}=\frac{0.53+0.28+0.16+0.19+0.25+0.31+0.53+0.52+0.51+0.50+0.50}{11}≐0.39$$


**Figure S2**. Schematic illustrating *P. aeruginosa* growth (grey) on microplates in microdilution checkerboard assay. Minimum inhibitory concentration for each antimicrobial is determined (blue). FICs are calculated for various combinations antibiotic-GCN/Ag nanohybrid inhibiting bacterial growth and at the same time minimal FIC (green), maximal FIC (red) and average FIC (FIC_AVR_) are highlighted and the resulting effect is described (SYN – synergy, PSY – partially synergy, ADD – additive effect).

**Figure S3**. Schematic illustrating *E. kobei* growth (grey) on microplates in microdilution checkerboard assay. Minimum inhibitory concentration for each antimicrobial is determined (blue). FICs are calculated for various combinations antibiotic-GCN/Ag nanohybrid inhibiting bacterial growth and at the same time minimal FIC (green), maximal FIC (red) and average FIC (FIC_AVR_) are highlighted and the resulting effect is described (SYN – synergy, PSY – partially synergy, ADD – additive effect).

*E. coli* CE 5556

|  | **AMP** | **AMS** | **CZL** | **CRX** | **CXT** | **GEN** | **COT** | **COL** | **OXO** | **OFL** | **TET** | **AZT** |
| --- | --- | --- | --- | --- | --- | --- | --- | --- | --- | --- | --- | --- |
| **BP** | **8** | **8** | **4** | **8** | **8** | **2** | **16** | **2** | **8** | **0,5** | **2** | **4** |
| E. coli | >64 | 16 | >64 | >64 | 8 | 128 | >256 | 0,5 | >32 | >8 | >64 | >16 |
|  | **PIP** | **PPT** | **CPR** | **CTX** | **CTZ** | **CPM** | **CPS** | **MER** | **CIP** | **TIG** | **TOB** | **AMI** |
| **BP** | **16** | **16** | **8** | **2** | **4** | **4** | **8** | **8** | **0,5** | **1** | **2** | **8** |
| E. coli | >128 | 32 | >64 | >16 | 32 | >16 | 16 | 0,1 | 64 | 0,1 | >32 | 8 |

*P. aeruginosa* 21425

|  | **PIP** | **PPT** | **AZT** | **MER** | **CTZ** | **CPR** | **CPM** | **GEN** | **AMI** | **COL** | **OFL** | **CIP** |
| --- | --- | --- | --- | --- | --- | --- | --- | --- | --- | --- | --- | --- |
| **BP** | **16** | **16** | **16** | **8** | **8** | **8** | **8** | **4** | **16** | **2** | **0,5** | **0,5** |
| P. aeruginosa | 128 | 128 | 32 | 16 | 8 | >32 | 16 | 8 | 0,5 | 1 | >8 | 16 |

*E. kobei* 3683

|  | **AMP** | **AMS** | **CZL** | **CRX** | **CXT** | **GEN** | **COT** | **COL** | **OXO** | **OFL** | **TET** | **AZT** |
| --- | --- | --- | --- | --- | --- | --- | --- | --- | --- | --- | --- | --- |
| **BP** | **8** | **8** | **4** | **8** | **8** | **2** | **16** | **2** | **8** | **0,5** | **2** | **4** |
| E.kobei | 16 | 16 | 16 | 16 | 16 | 2 | 2 | 64 | 0,25 | 0,06 | 4 | 0,25 |
|  | **PIP** | **PPT** | **CPR** | **CTX** | **CTZ** | **CPM** | **CPS** | **MER** | **CIP** | **TIG** | **TOB** | **AMI** |
| **BP** | **16** | **16** | **8** | **2** | **4** | **4** | **8** | **8** | **0,5** | **1** | **2** | **8** |
| E.kobei | 2 | 2 | 0,5 | 0,125 | 0,125 | 0,125 | 0,5 | 0,063 | 0,063 | 0,063 | 0,25 | 1 |

**Figure S.4** MIC (mg/l) values of used strains, BP = MIC breakpoints mg/L according to EUCAST, AMP – ampicillin, AMS -ampicillin/sulbactam, CZL – cefazoline, CRX – cefuroxime, CXT – cefoxitine, GEN – gentamicin, COT – cotrimoxazole, COL – colistin, OXO – oxolin acid, OFL – ofloxacin, TET – tetracycline, AZT – aztreonam, PIP – piperacillin, PPT – piperacillin/tazobactam, CPR – cefoperazone, CTX – cefotaxime, CTZ – ceftazidime, CPM – cefepime, MER – meropenem, CIP – ciprofloxacin, TIG – tigecycline, TOB - tobramycin, AMI – amikacin
